# Structure-Based Identification of JAK1-Selective Candidates Using Ensemble Docking and Interaction Analysis

**DOI:** 10.3390/ph19050709

**Published:** 2026-04-30

**Authors:** Nicoleta Stoian, Sorin Avram, Liliana Halip

**Affiliations:** Department of Computational Chemistry, “Coriolan Drăgulescu” Institute of Chemistry Timișoara, Romanian Academy, 24 Mihai Viteazu Avenue, 300223 Timișoara, Romania

**Keywords:** JAK1 inhibitors, selectivity, autoimmune diseases, molecular docking, structure-based drug design, kinase fingerprinting, scaffold analysis

## Abstract

**Background/Objectives**: Selective inhibition of JAK1 remains a major challenge in cytokine-signaling therapeutics due to the high structural similarity of the JAK family. Here, we present an integrated computational framework that combines large-scale binding-site conformational analysis, ensemble docking, and protein–ligand interaction fingerprinting (PLIF) to elucidate the structural determinants of JAK1 selectivity and prioritize JAK1-biased scaffolds. **Methods**: A curated set of JAK1 and JAK2 catalytic-domain structures was clustered to capture binding-site diversity, and representative conformers were evaluated using >2300 annotated ligands. Docking performance was assessed via AUC, early enrichment metrics, and structural pose validation against experimentally resolved complexes. The workflow was subsequently applied to a library of ~6000 drug-like compounds to prioritize candidates with predicted JAK1 preference. **Results**: Across the ensemble, the most predictive features reliably separated active from inactive ligands (AUC = 0.78–0.82) and captured subtle, systematic rank shifts supporting the reported JAK1 bias. Interaction fingerprint analysis revealed a conserved hinge-binding motif required for potency, alongside a JAK1-enriched hotspot adjacent to Glu aD.55 that contributes to isoform discrimination. Applied to a library of ~6000 drug-like molecules, the workflow yielded 174 candidates predicted to exhibit preferential JAK1 recognition and reduced JAK2 engagement. **Conclusions**: These findings define the structural and physicochemical features underlying JAK1 selectivity and illustrate how ensemble-based modeling can guide the discovery of next-generation selective kinase inhibitors.

## 1. Introduction

Autoimmune diseases, including psoriasis and rheumatoid arthritis (RA), affect millions of individuals worldwide, placing a major burden on healthcare systems [1,2,3]. These conditions are characterized by chronic inflammation driven by dysregulated immune signaling, leading to tissue damage, functional impairment, and reduced quality of life. Current therapeutic strategies frequently target key inflammatory proteins like tumor necrosis factor-α (TNF-α) [4,5], interleukin-6 (IL-6) [6], and interleukin-17 (IL-17) [7,8] using monoclonal antibodies and other biologic drugs. While these treatments have brought significant benefits, they are associated with high costs, potential immunogenicity, and variable patient response.

Recent studies have identified Janus kinase 1 (JAK1) as a clinically relevant therapeutic target for these autoimmune conditions [9,10,11]. The JAK family of cytoplasmic tyrosine kinases (JAK1, JAK2, JAK3, and TYK2) plays a central role in cytokine-mediated signaling through the JAK-STAT pathway, regulating gene expression programs involved in immune function and inflammation [12,13]. JAK1 is involved in signaling of multiple cytokines central to autoimmune pathogenesis, including interferon-γ, IL-6, and IL-2, enabling broad anti-inflammatory effects through a single small-molecule intervention [14,15]. Small-molecule JAK inhibitors have demonstrated clinical efficacy, but the first-generation agents (e.g., tofacitinib, baricitinib) lack isoform selectivity and therefore inhibit JAK2 to a clinically meaningful extent [16,17,18]. Because JAK1 and JAK2 share highly conserved catalytic domains, most existing inhibitors exhibit cross-reactivity [19], and JAK2 inhibition is strongly associated with hematopoietic suppression and dose-limiting toxicities such as anemia and thrombocytopenia [20,21,22,23]. Achieving robust JAK1 selectivity therefore remains essential for maximizing therapeutic benefit while minimizing dose-limiting toxicities.

Recent efforts to improve kinase selectivity have focused on structure-based drug design targeting unique binding pockets, allosteric modulation of regulatory domains, and exploitation of subtle conformational differences between highly similar active sites. These strategies represent the current frontier in developing next-generation JAK1-selective therapeutics for autoimmune diseases. Although several second-generation inhibitors demonstrate preferential JAK1 activity, their selectivity profiles remain dependent on the assay conditions and are often modest when quantified using biochemical metrics such as ΔpIC_50_. In this context, ΔpIC_50_ values of 0.5–1.5 (approximately 3–50-fold selectivity) are commonly reported, whereas higher levels of selectivity are rarely achieved or consistently validated across experimental systems [24,25,26]. Truly selective molecules (ΔpIC_50_ ≥ 2) remain rare and often lack independent validation across assay formats [27,28]. Overall, while progress toward JAK1-preferential inhibition has been significant, achieving true isoform exclusivity over JAK2 continues to be an ongoing challenge. To ensure clarity throughout this study, we define “selectivity” strictly in the context of experimentally validated isoform discrimination (e.g., quantified by ΔpIC_50_). In contrast, terms such as “preference” and “bias” are used to describe computationally predicted tendencies for a ligand to rank more favorably for JAK1 over JAK2, which indicate structural compatibility trends but do not imply a confirmed quantitative experimental selectivity margin.

Although JAK selectivity has been widely investigated, computational studies have typically relied on individual crystal structures or on methods that sample a narrower portion of the conformational landscape. Molecular dynamics simulations have also provided valuable insights into JAK family behavior, offering a powerful way to explore protein flexibility, but they require substantial computational resources and are not always practical for large comparative analyses. In this context, the structural factors that differentiate JAK1 from JAK2 can be examined through multiple methodological perspectives, and our study contributes an additional viewpoint by analyzing these residue-level differences using a curated multi-structure ensemble and comparative protein–ligand interaction analysis.

**Figure 1:**
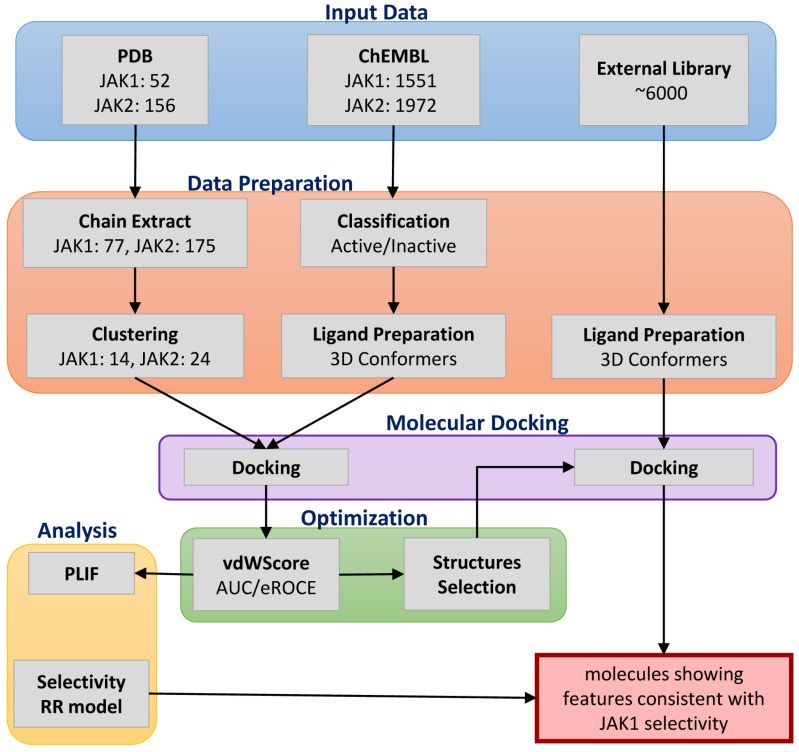
Overview of the computational strategy used in this study to prioritize compounds with potential JAK1 selectivity. The diagram summarizes the specific combination of structural ensemble selection, docking-based ranking, and comparative analysis applied in our JAK1/JAK2 investigation.

This study integrates conformational clustering, ensemble docking, comparative protein–ligand interaction analysis, and selectivity-oriented ranking to highlight structural patterns associated with JAK1 preference. We then applied this tailored methodology to a focused library of 6000 screening compounds to identify scaffolds with potential JAK1-selective features. An overview of the methodological steps used in this study is provided in Figure 1.

## 2. Results and Discussions

### 2.1. Structural Overview of JAK1 and JAK2 Catalytic Domains

**Figure 2:**
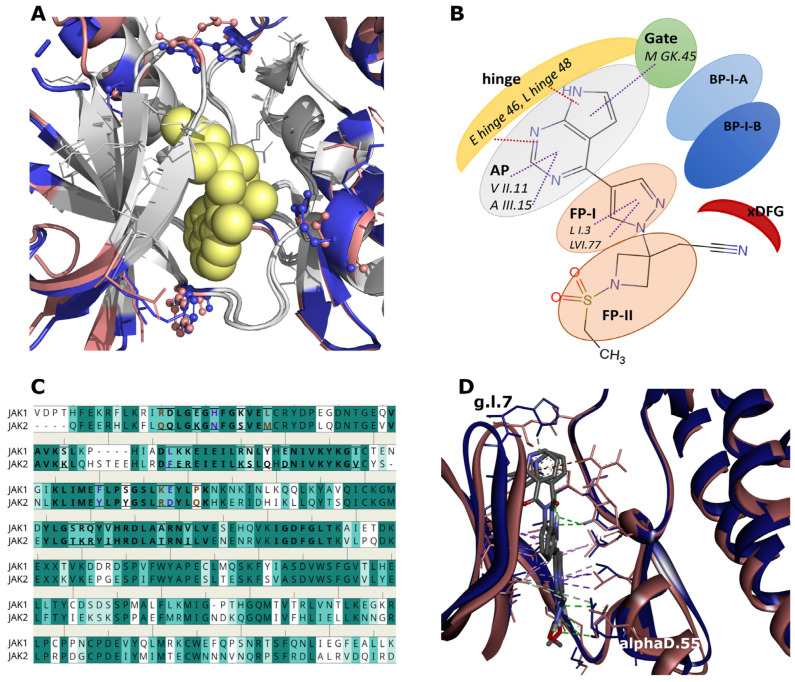
(**A**) Overlay of the JAK1 (blue) and JAK2 (salmon) catalytic domains. KLIFS binding-site residues are shown in gray. Differing residues are displayed as: lines—all differing residues; sticks and balls—differing residues with side chains oriented toward the FPI site; sticks—oriented toward the FPI site but positioned farther from the cavity. The yellow surface marks the FPI-binding region. (**B**) Schematic diagram of front and back pockets of JAK1 with bound baricitinib (AP: adenine pocket, FP: front pocket, BP: back pocket). (**C**) Sequence alignment of the JAK1 and JAK2 catalytic domains, with KLIFS-defined residues in bold, sequence differences boxed, and residues oriented toward the FPI site highlighted in violet or amber depending on proximity. Residue background colors reflect sequence identity: dark teal for identical positions, lighter teal for decreasing similarity, and white for no similarity. (**D**) Lead compound bound to JAK1 (PDB: 6SMB, blue) and JAK2 (salmon). Interactions are shown as dashed lines: green—H-bonds; purple—hydrophobic; orange—salt bridges.

Among the 85 KLIFS-defined positions describing the canonical kinase binding site [29,30], JAK1 and JAK2 differ at 26 residues, mainly distributed across the N-(16) and C-terminal (10) lobes. Only four substitutions directly face the ligand-binding cavity (Phe/Tyr at hinge.47, His/Asn at g.l.7, Leu/Phe at a.l.21, and Glu/Asp at aD.55), while four additional differences (Lys/Arg aD.54, Pro/Gln aD.58, Arg/Gln I.1, and Asp/Glu II.13) lie near the pocket entrance. (Figure 2A). The remaining variations are solvent-exposed and unlikely to influence ligand binding. Peripheral positions such as aD.54, aD.58, and I.1 were examined but did not show consistent selectivity-related interaction patterns.

The four substitutions inside the pocket are conservative, yet capable of subtly modulating local interactions. At hinge.47, Phe/Tyr preserves aromaticity while introducing a potential hydrogen-bond donor. At g.l.7, His/Asn removes an ionizable imidazole, altering polar and proton-transfer interactions. The Leu/Phe change at a.l.21 adds an aromatic ring in back-pocket I (BP-I), enabling π–π contacts while maintaining hydrophobic packing, an element previously exploited in selective kinase inhibitor design [31,32] (Figure 2B). The Glu/Asp substitution at aD.55 retains charge but shortens the side chain, subtly shifting local electrostatics. Collectively, these variations do not alter the global fold but fine-tune the physicochemical microenvironment, particularly within front-pocket I (FP-I) (Figure 2C). Their functional relevance is illustrated by the co-crystal structure of a lead compound used in the development of the JAK1-selective inhibitor golidocitinib [27]. In JAK1, the ligand forms stabilizing contacts with His g.l.7 and Glu aD.55. In the JAK2 model, both interactions are lost: Asn replaces His at g.l.7, removing a proton-exchange-capable side chain, while Asp replaces Glu at aD.55, shortening the distance and eliminating a weak C–H···O hydrogen bond (Figure 2D). Although individually subtle, these effects accumulate and likely contribute to the experimentally observed JAK1 selectivity.

### 2.2. Conformational Space of JAK1 and JAK2 Binding Site

**Figure 3:**
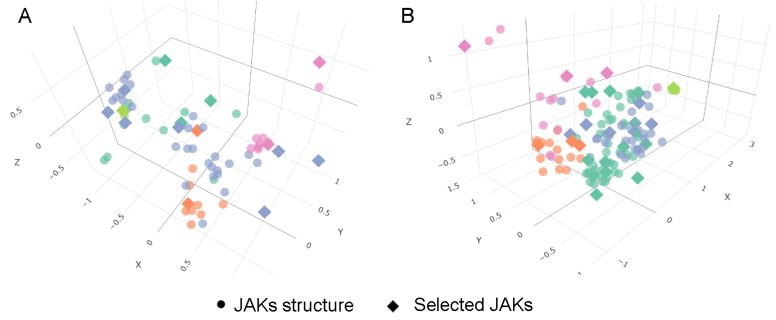
Conformational clustering of JAK1 (**A**) and JAK2 (**B**) binding sites. Distinct clusters are color-coded to illustrate structural variability around the ligand-binding pocket. Representative structures for each cluster are marked with diamond symbols.

To avoid redundancy and ensure meaningful coverage of binding-site flexibility, we constructed a reduced ensemble of representative conformations for JAK1 and JAK2. Because structurally similar conformations are often deposited as distinct entries, directly using all available structures in docking would lead to overrepresentation of certain states without increasing conformational diversity. To address this, structural variability within the KLIFS-defined binding site was quantified using heavy-atom RMSD, and conformations were grouped by complete-linkage clustering into distinct conformational families (Figure 3). Representative structures (one to three per cluster) were then selected based on cluster population (Table S1 in Supplementary Materials), ensuring that the final ensemble captures the full extent of observed conformational space while minimizing redundancy.

To further refine the selection, representatives were prioritized using LBI (Ligand Binding Index), which favors conformations with demonstrated ability to reproduce crystallographic ligand poses [33]. This step ensured that the retained structures are not only structurally diverse but also relevant for docking applications.

The resulting ensembles comprise 15 conformations for JAK1 and 24 for JAK2 (Figure 3), reflecting the broader structural variability observed for JAK2. This curated set provides a balanced representation of binding-site conformations suitable for downstream docking and selectivity analysis, without bias from overrepresented structural states. Intra-cluster RMSD statistics and selection metrics are summarized in Table S1. This approach ensures that docking results reflect genuine conformational diversity rather than artifacts of structural database redundancy.

### 2.3. Screening Guided Prioritization of Protein Structures

To identify the JAK1 and JAK2 representative structures having the best ability to discriminate between active and inactive ligands, we assembled two balanced benchmark datasets (Figure S1 in Supplementary Materials). Each set contained several hundred experimentally confirmed actives complemented by a comparable number of inactives, providing a robust basis for assessing screening performance. A substantial fraction of compounds is shared between the two datasets, reflecting the high chemical similarity of JAK inhibitors and enabling direct comparison of structure-dependent ranking behavior across kinases. Consistent with this overlap, global descriptor analysis shows that active ligands for both targets exhibit highly similar physicochemical profiles (Figure S2 in Supplementary Materials), underscoring the intrinsic challenge of achieving isoform selectivity.

To probe sensitivity to subtle selectivity signals, a subset of literature-reported [24,25,26] JAK1-biased inhibitors having selectivity margins of ≤1.5 log units was included (Table S2 in Supplementary Materials). These compounds serve as internal benchmarks for assessing whether docking can recover weak but meaningful preference trends. Together, these datasets provide a robust framework for validating the screening protocol and prioritizing protein conformations based on their ligand discrimination performance.

#### Virtual Screening Validation and Rank Consistency

To assess the reliability of the virtual screening workflow, we implemented a two-level validation strategy: (i) evaluation of docking performance using balanced active/inactive datasets, and (ii) analysis of rank consistency across kinases for compounds with experimentally confirmed dual activity.

**Figure 4:**
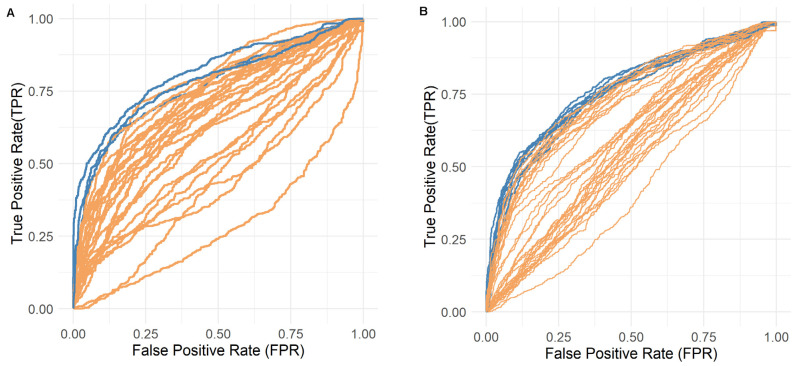
ROC curves for JAK1 (**A**) and JAK2 (**B**), with top-performing runs in terms of metrics shown in blue. Full confusion-matrix statistics, together with precision, recall, and F1 scores, evaluated across multiple cutoff thresholds, are reported in the Supplementary Materials (Table S3).

Each representative JAK1 and JAK2 structure was used as an independent docking target. Ligands were ranked using multiple docking scoring functions, and performance was assessed using several complementary metrics capturing both global discrimination and early recognition. While several scoring functions performed better than random, the van der Waals interaction score (vdWs) consistently yielded superior performance across most structures, suggesting the steric complementarity is a primary determinant of ligand discrimination for both kinases. Although multiple conformations achieved comparable AUC values, structure selection was primarily guided by early enrichment metrics (eROCE and EF1%), which better reflect applied screening performance. Early enrichment analysis shows consistent performance across both kinases, with enrichment factors around ~2.0 at 1–10% cutoffs and comparable precision–recall trade-offs, confirming that active compounds are preferentially ranked within the top fraction of screened molecules (Table S3 in Supplementary Materials). Applying combined criteria (AUC ≥ 0.70 and strong early enrichment) resulted in the selection of four JAK1 and nine JAK2 conformations for downstream analysis, representing the most predictive regions of the conformational landscape (Figure 4).

**Figure 5:**
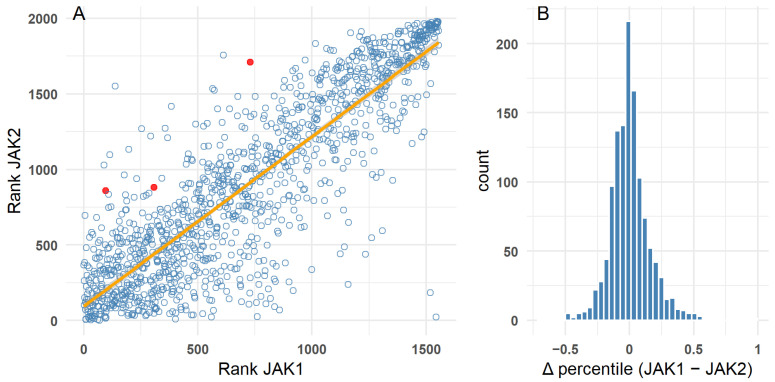
(**A**) Rank–rank scatter plot (red: itacitinib, filgotinib, upadacitinib); (**B**) Distribution of percentile differences (ΔJAK2 − JAK1).

To further evaluate model consistency, docking-derived ligand rankings were compared between JAK1 and JAK2. Scores were converted to normalized percentile ranks to enable direct comparison. Rank-rank analysis revealed strong agreement, supported by Spearman’s ρ = 0.85 and Kendall’s τ = 0.66 (*p* < 0.001), indicating substantial concordance between the two targets (Figure 5). This high correlation is the expected baseline given the extensive structural conservation between the JAK1 and JAK2 catalytic domains, against which selective deviations are measured. The distribution of percentile differences (ΔJAK2 − JAK1) was centered near zero and approximately symmetric, suggesting no systematic bias toward either kinase. Notably, JAK1-biased inhibitors (e.g., itacitinib, filgotinib, upadacitinib) consistently ranked higher for JAK1 than JAK2, demonstrating that the workflow can capture subtle selectivity trends despite the high structural similarity of the binding sites (red in Figure 5).

Together, the combined global discrimination metrics, early enrichment analyses, and cross-kinase consistency results demonstrate that the virtual screening workflow is sensitive to binding-site conformational variation, capable of recovering known enrichment patterns, and quantitatively robust. These findings validate the adopted multi-structure docking strategy and provide a foundation for subsequent analyses of binding-interaction fingerprints and inhibitor selectivity.

### 2.4. Binding Interaction Fingerprints and Selectivity Determinants

To quantify interaction features underlying JAK1–JAK2 discrimination, we analyzed Glide-derived docking score components for the subset of shared active ligands. Van der Waals (vdW) and electrostatic contributions were compared using paired statistical analysis. The vdW interactions show a consistent shift toward more favorable values in JAK1 (Wilcoxon test, *p* = 2.2 × 10^−16^), whereas electrostatic contributions largely overlap and are not significantly different (*p* = 0.095). These reported *p*-values remain significant after accounting for multiple comparisons using a standard Bonferroni correction (adjusted α = 0.025). These results indicate that hydrophobic packing is the primary contributor to ligand discrimination within this docking framework, while electrostatic interactions remain largely conserved. The preferential vdW stabilization in JAK1 further correlates with improved pose stability across its structural ensemble, suggesting that conformational variability modulates selectivity by influencing hydrophobic complementarity. Importantly, while the vdW energy term is an empirical scoring component, this finding is independently supported by the structural biology of the binding sites—specifically the Leu/Phe substitution at a.l.21 and the Glu/Asp substitution at aD.55, which directly alter the hydrophobic cavity—as well as by the geometric PLIF analysis described below.

**Figure 6:**
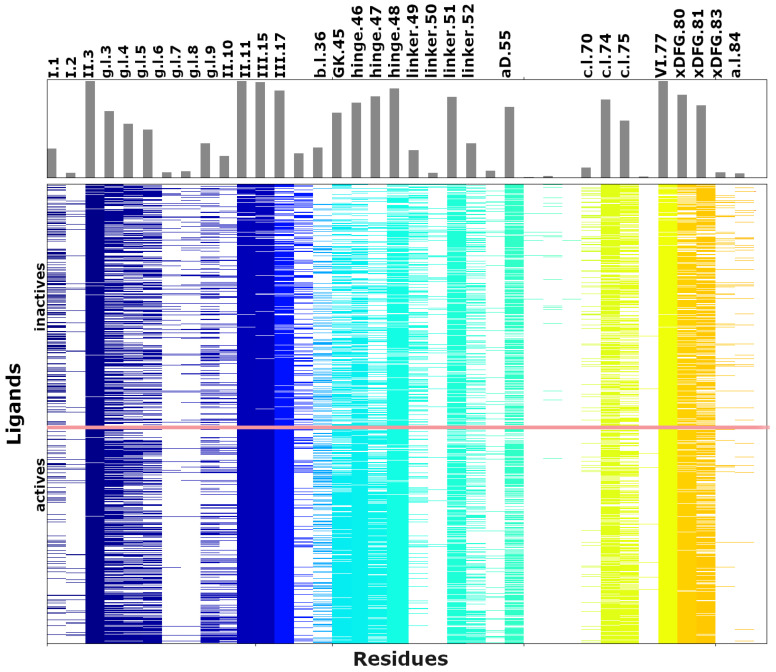
Interaction fingerprint for the selected dataset of JAK1 inhibitors. To generate the interaction fingerprint, compounds were ranked according to their experimentally determined activity, with active compounds displayed below the red line and inactive inhibitors on top of the red line.

To gain structural insight into these trends, protein–ligand interaction fingerprints were analyzed across representative conformations. Two complementary patterns emerge: (i) conserved interactions associated with ligand potency, and (ii) non-conserved contacts that may contribute to selectivity. Active ligands consistently form a canonical hinge-binding motif involving two backbone hydrogen bonds (hinge.46 and hinge.48), which anchors the heterocyclic core within the ATP-binding site. This observation is in agreement with literature data and emphasizes the importance of hinge hydrogen bonding as a key determinant of potent kinase inhibition [34,35]. Inactive compounds rarely reproduce this motif, indicating non-productive binding orientations. (Figure 6).

Beyond polar contacts, active ligands establish an extended hydrophobic network within the adenine pocket (Leu I.3, Val II.11, Ala III.15, Leu VI.77). While part of these contacts (e.g., Leu I.3, Leu VI.77) are common to both active and inactive compounds due to structural constraints, others such as Ala III.15 or the gatekeeper Met GK.45 are enriched in active ligands, supporting optimal packing near the hinge region. Additional contacts with Lys II.10 and Val bI.36 further differentiate active compounds, whereas interactions involving Arg I.1, Pro linker.50, Asp cI.70, Gly xDFG.83, and Leu aI.84 are more frequently associated with inactive binding modes. Importantly, many of these interactions occur at positions that differ subtly between JAK1 and JAK2, indicating that selectivity arises not from distinct binding modes but from fine-tuning of a shared interaction network. Small variations in side-chain composition and conformational flexibility appear sufficient to bias hydrophobic packing, consistent with the vdW-driven discrimination observed in the scoring analysis.

The molecular determinants of JAK1 inhibitor potency largely reflect the conserved kinase-binding paradigm, including dual hinge hydrogen bonds, hydrophobic packing within the adenine pocket, and stabilization by the catalytic and DFG-region interaction. These shared features explain the similar physicochemical profiles and binding modes observed for JAK1 and JAK2 inhibitors (Figure S2).

In contrast, selectivity arises from a limited number of non-conserved residues that modulate local interactions. Among these, Glu aD.55 in JAK1 emerges as the most consistent selectivity hotspot. Its longer side-chain, relative to the corresponding Asp in JAK2, enables direct or water-mediated hydrogen-bonding interactions that are less accessible in JAK2 (Figure S3), providing a subtle but reproducible stabilization advantage. Other variable positions (hinge.47, g.l.7, and a.l.21) primarily influence local binding geometry in a context-dependent manner rather than acting as dominant selectivity drivers. In addition, increased flexibility of the JAK1 P-loop facilitates accommodation of bulkier substituents, whereas the more constrained JAK2 loop can introduce steric penalties for similar ligands. Peripheral residues, such as Lys aD.54, further contribute by supporting interactions that are less favorable in JAK2.

**Figure 7:**
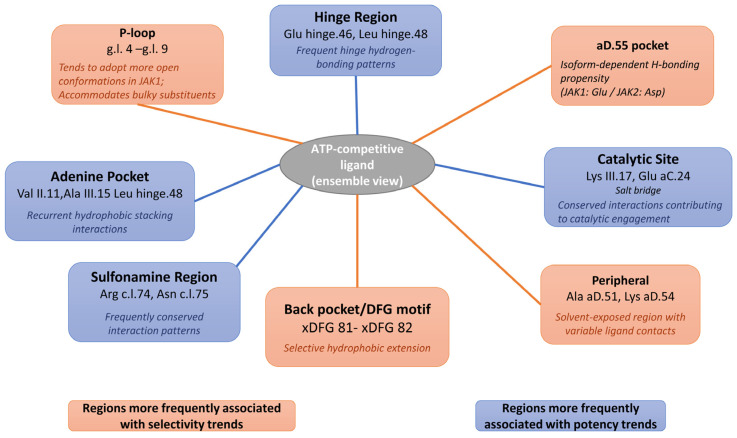
Schematic representation of key interaction regions contributing to ATP-competitive ki-nase inhibition. The diagram summarizes frequently observed contact zones based on ensemble docking and statistical interaction analysis.

Together, these observations support a dual design strategy for JAK1-selective inhibitors: a conserved anchoring core that engages conserved hinge and catalytic features to secure potency, and tailored substituents that exploit Glu aD.55 and P-loop flexibility to discriminate against JAK2 (Figure 7). Clinically relevant compounds such as golidocitinib exemplify this strategy, achieving high selectivity while maintaining nanomolar affinity. Consistent with these structural insights, JAK1-selective compounds are enriched among top-ranked ligands in JAK1 screens but do not occupy the highest-ranking percentiles in JAK2, reflecting their reduced (but measurable) affinity for the latter. This behavior further supports the ability of the docking workflow to capture subtle selectivity differences.

### 2.5. Prospective Virtual Screening on an External Library

The validated docking framework was applied to an external library of ~6000 bioactive small molecules with documented bioactivity potential, to identify candidates with preferential predicted affinity for JAK1 over JAK2. Compounds were ranked using the selected ensemble of high-performing conformations, and the top 5% (~300 molecules) were retained for further analysis. Selectivity was assessed using the relative rank (RR) difference, where negative values indicate preferential ranking in JAK1. After filtering out compounds with RR > 0, a final set of 174 JAK1-enriched candidates was obtained (Table S4), representing the most promising subset for selectivity-driven prioritization. Analysis of docking pose consistency revealed that high-ranking ligands adopt stable binding modes across the JAK1 ensemble, with average pairwise RMSD values typically below 2.0 Å. In contrast, the same compounds exhibit greater pose variability in JAK2, suggesting reduced compatibility with its conformational landscape (Table S5). This observation is consistent with the vdW-driven selectivity trends identified earlier and supports the role of binding mode stability in JAK1 preference.

#### 2.5.1. Target-Annotation-Based Validation

To further assess the relevance of the prioritized candidates, we examined their known biological annotations as an orthogonal validation of the screening workflow. Among the 174 JAK1-enriched compounds, 115 (~66%) have documented activity against kinase targets, indicating a strong enrichment of kinase-relevant chemotypes within the selected subset.

**Figure 8:**
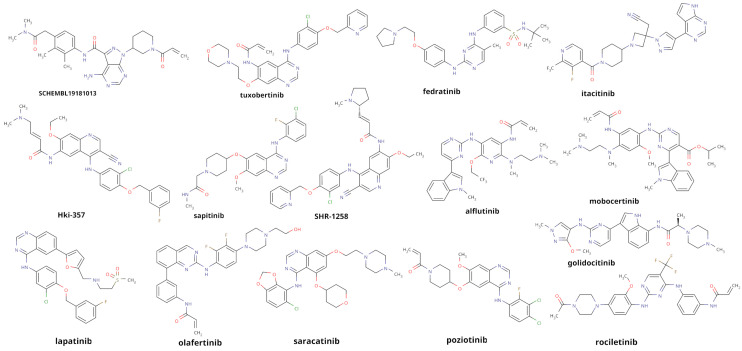
Top-scoring ligands from virtual screening with reported kinase inhibition data.

Importantly, 15 compounds are associated with the JAK/STAT signaling axis (Figure 8), providing additional support that the screening strategy captures chemical features consistent with JAK pathway modulation. Given that the initial library comprises structurally diverse bioactive molecules spanning multiple target classes (e.g., GPCR ligands, ion channel modulators, enzyme inhibitors), such enrichment is unlikely to arise from random selection. This observation therefore serves as a secondary validation layer, suggesting that the docking-driven prioritization does not merely identify high-scoring compounds but selectively enriches molecules belonging to biologically relevant chemical space. At the same time, it is important to note that pathway-level annotations may reflect indirect or upstream interactions and do not necessarily imply direct inhibition of JAK1 or JAK2. In this context, structure-based metrics such as the RR provide an essential filter to refine target specificity.

#### 2.5.2. ADME-Tox Profiling of Prioritized Candidates

To evaluate the developability of the prioritized compounds, in silico ADME pro-filing was performed using SwissADME [36]. The results indicate that the majority of compounds exhibit moderate bioavailability, with approximately two-thirds satisfying common drug-likeness criteria. Predicted properties such as CYP450 inhibition, P-gp substrate behavior, and reduced gastrointestinal absorption are consistent with known characteristics of kinase inhibitors [37,38], which frequently deviate from classical drug-likeness rules. Accordingly, these features were not used as exclusion criteria but rather as guidance for future optimization and experimental evaluation. Overall, the ADME profiles support the chemical plausibility of the prioritized candidates and are consistent with the physicochemical space typically occupied by ATP-competitive kinase inhibitors.

#### 2.5.3. Relevance and Integration with Selectivity Analysis

The prioritized compounds are consistent with the structural determinants identified in the structural analysis presented in the previous section. Ligands with negative RR values retain canonical hinge interactions and hydrophobic packing within the adenine pocket while incorporating substituents compatible with the JAK1-specific Glu aD.55 environment and increased P-loop flexibility. The enrichment of kinase-like scaffolds further supports that the docking-driven filtering effectively captures chemotypes capable of forming the interaction fingerprint motifs characteristic of selective JAK1 inhibitors.

These results support a model in which selectivity arises from incremental optimization of shared interaction motifs rather than the formation of unique binding modes. While RR values do not directly translate into quantitative selectivity, they provide a useful framework for identifying compounds with preferential JAK1 engagement.

Experimental validation across a broader kinase panel will be required to confirm selectivity and assess off-target effects. Nonetheless, the present strategy provides a robust basis for prioritizing candidates and guiding hypothesis-driven optimization.

### 2.6. Limitations

While the presented computational workflow provides a robust framework for prioritizing JAK1-selective candidates over JAK2, it is important to acknowledge inherent limitations. First, all findings are based on in silico docking and interaction analyses. Although the workflow demonstrates consistent discrimination and mechanistic coherence, experimental validation is required to confirm binding affinities and selectivity profiles. Biochemical and cellular assays will be necessary to establish the true inhibitory potency of the prioritized compounds. Second, selectivity assessment relies on relative ranking rather than direct estimation of binding free energies. While RR provides a useful comparative framework, it cannot be directly translated into quantitative selectivity metrics such as ΔIC_50_. As a result, the identified compounds should be interpreted as candidates with preferential binding trends rather than definitive isoform-selective inhibitors.

The external screening library, although designed to maximize structural diversity, remains limited to approximately 6000 compounds. The enrichment of kinase-related chemotypes among the prioritized hits supports the effectiveness of the approach; however, broader exploration of chemical space may reveal additional JAK1-selective scaffolds. Also, while the use of multiple protein conformations captures static structural variability, the present study does not explicitly account for dynamic effects such as binding stability and conformational transitions over time. These aspects would require molecular dynamics simulations or free-energy calculations to further refine selectivity predictions. Overall, these limitations highlight the need for experimental validation and suggest opportunities for future work, including expanded chemical libraries and dynamic modeling approaches.

## 3. Materials and Methods

### 3.1. Selection of Active and Inactive Compound Sets Targeting JAK1 and JAK2

Data for approved JAK1 and JAK2 inhibitors (targets CHEMBL2835 and CHEMBL2971) were extracted from ChEMBL database [39], along with compounds annotated from biochemical assays (labels as “B”). Only entries with available chemical structures and SMILES codes were retained for further analysis. Biological assay records were filtered by standard type to ensure consistency. Compounds were classified as “active” if they exhibited a pChEMBL value of >7 or a standard value ≤ 100 nM, and as “inactive” if they showed a pChEMBL ≤ 5.5 or a standard value ≥ 10 microM. JAK1 dataset comprises 1551 molecules, including 694 actives and 857 inactives, while JAK2 dataset contains 1972 molecules, of which 788 were active and 1184 inactive. The two datasets shared 597 active and 626 inactive compounds, providing a consistent subset for cross-dataset comparisons and validation.

### 3.2. Protein Selection for Docking

Protein–ligand complex structures of human JAK1 and JAK2 were retrieved from the Protein Data Bank (PDB) (Table S6 in Supplementary Materials) [40] using their corresponding UniProt identifiers (P23458 for JAK1 and O60674 for JAK2). Of the 52 JAK1 and 156 JAK2 multi-chain crystal structures collected initially, only those containing the catalytic kinase domain were retained. Individual protein chains were isolated from each crystal structure, yielding 77 JAK1 and 175 JAK2 monomers. Structural preprocessing involved removing crystallographic components irrelevant for ligand binding (e.g., buffer molecules, low-molecular-weight additives, or cofactors) while all co-crystallized ligands (ATP-site or allosteric) were preserved, serving as references for defining binding pockets.

For each JAK, monomers were subsequently aligned to enable direct structural comparison. Binding sites were defined as all residues within 5 Å of the co-crystallized ligand. Conformational similarity between binding sites was quantified by pairwise root-mean-square deviation (RMSD) calculations performed on all heavy atoms of the binding-site residues. The RMSD distance matrix was analyzed using hierarchical agglomerative clustering (complete linkage) as implemented in “cluster” package in R version 4.4.1 [41], resulting in the identification of five distinct and well-separated structural clusters for each JAK. (Figure S4 in Supplementary Materials). After hierarchical clustering of the RMSD distance matrix, representative structures were selected using a two-step strategy aimed at maximizing intra-cluster coverage while minimizing redundancy. For each cluster, the medoid structure was selected as the primary representative. In clusters with larger population size or increased internal dispersion, additional representatives were chosen iteratively by selecting the structure with the maximum RMSD distance from the previously selected representatives within the same cluster.

This approach ensured that selected structures were spatially well separated and collectively representative of the conformational variability captured by each cluster. For visualization purposes, the RMSD distance matrix was further projected into three dimensions using classical multidimensional scaling (MDS), providing an intuitive low-dimensional representation of the same distance relationships captured by dendrogram. The MDS projection does not introduce additional assumptions and was used solely to visualize structural diversity. Following spatial selection, candidate representatives were evaluated using the Ligand Binding Index (LBI) [33] to assess their suitability for docking. The Ligand Binding Index (LBI), originally validated on the CASF-2016 benchmark encompassing diverse protein target classes, was used as a target-agnostic criterion to prioritize docking-competent conformations. In cases where a medoid or distance-based representative exhibited poor docking fidelity, it was replaced by a neighboring structure that preserved both cluster representativeness and docking performance. A final dataset of 15 JAK1 and 24 JAK2 structures was obtained and used as templates for subsequent virtual screening.

### 3.3. Molecular Docking Protocol

Protein structures were prepared with the Protein Preparation Wizard (Schrödinger, LLC, New York, NY, USA) [42] by assigning bond orders, adding hydrogens, optimizing hydrogen-bonding networks, and removing crystallographic components not involved in ligand recognition. Ligands were processed with LigPrep (Schrödinger, LLC, New York, NY, USA) [43] to generate 3D conformations, enumerate relevant ionization states at pH 7.4, and minimize geometries using the OPLS force field. Receptor grids were centered on the ATP-binding site and sized so that all relevant subpockets were accessible during docking. Molecular docking was performed with Glide (Schrödinger, LLC) [44] in standard precision (SP) mode. It should be noted that rigid-receptor docking was employed; while the use of a multi-structure ensemble accounts for pre-existing static conformational diversity, dynamic induced-fit effects upon ligand binding were not explicitly modeled. For each ligand, multiple poses were scored using GlideScore and all associated scoring components. During benchmarking on the JAK1/JAK2 activity datasets, the van der Waals (vdW) energy term consistently showed the best ability to discriminate active from inactive compounds. However, vdW enrichment should not be interpreted as a physical decomposition of binding free energy, but rather as an empirical indicator of steric complementarity within the sampled conformational ensemble.

### 3.4. Evaluation of Virtual Screening Performance

The performance of each docking protocol and scoring component was evaluated using a set of complementary metrics designed to assess both global ranking quality and early enrichment behavior. Discrimination between active and inactive compounds was quantified using receiver operating characteristic (ROC) curve analysis [45], in which true positive rates (TPR) were plotted against false positive rates (FPR) for a range of score thresholds. The corresponding area under the curve (AUC) was used as a global, threshold-independent measure of overall discriminative performance [45]. To more sensitively quantify the early recognition of active compounds—a critical requirement for virtual screening of large libraries—we also employed the exponential Receiver Operating Characteristic Enrichment (eROCE) metric [46]. Briefly, eROCE assigns exponentially decreasing weights to active compounds retrieved at higher FPRs, thereby emphasizing the quality of the top-ranked portion of the list. Higher eROCE values therefore correspond to binding-site conformations with improved early enrichment capability. eROCE was employed as the primary early recognition metric because it provides a continuous, threshold-independent evaluation of early enrichment and enables direct comparison across datasets of different sizes and class imbalance. In addition to eROCE, early enrichment performance was further characterized using confusion-matrix-derived metrics, including precision, recall, and F1 scores, computed at multiple top-ranked cutoffs (0.2%, 0.5%, 1%, 2%, 5%, and 10%). For each cutoff, true positives, false positives, and false negatives were calculated and aggregated across all receptor conformations included in the ensemble. Enrichment factors at a 1% cutoff (EF1%) were also computed to provide an intuitive and widely used measure of active compound enrichment among the highest-ranked fraction. All analyses were performed consistently for JAK1 and JAK2, ensuring unbiased comparative evaluation of docking performance and facilitating subsequent rank-based selectivity analyses.

### 3.5. Relative Ranking Score

To enable a direct comparison of docking outcomes between JAK1 and JAK2, a relative ranking score (RR) was defined. For each representative structure, all compounds in the corresponding JAK1 and JAK2 activity dataset were ranked according to their docking score, with rank 1 assigned to the best-scoring ligand. Because each kinase was docked against a different number of ligands, raw ranks were not directly comparable across JAK1 and JAK2. To address this, ranks were first normalized within each dataset by dividing the raw ranking position by the total number of ligands docked to that structure. This yielded normalized ranks, where lower values indicate better predicted binding affinity.

The relative ranking score was then calculated as in Equation (1):

RR= R_JAK1_ − R_JAK2_ (1)

where R_JAK1_ and R_JAK2_ denote the normalized ranks of the same compound against JAK1 and JAK2, respectively.

Values near zero indicate comparable affinity predictions for both kinases, as expected for non-selective compounds. Highly negative values reflect preferential recognition by JAK1 (better JAK1 rank), while highly positive values indicate a shift toward JAK2. The same RR formulation was subsequently applied to the external screening library, where the same ligand set was used for both JAK1 and JAK2, and normalization was not required. RR does not represent a quantitative measure of selectivity (such as ΔIC_50_ or ΔG), but a relative prioritization metric within a defined chemical space. Therefore, RR serves as a hypothesis-generating tool for prioritizing candidates for subsequent experimental testing, rather than a direct proxy for binding free energy. For the final hit selection, only compounds exhibiting substantial rank separation between JAK1 and JAK2 were retained, thereby excluding cases with marginal RR values unlikely to reflect meaningful selectivity.

### 3.6. External Screening Library for Prospective Hit Identification

The optimized virtual screening workflow was subsequently applied to an external library of ~6000 small molecules [47] intended for prospective identification of novel JAK1-selective inhibitors. This library consisted of approximately 2700 approved drugs and natural products, together with 3000 diversity-oriented synthetic compounds selected to maximize chemical space coverage. All molecules were processed using the same ligand preparation protocol as for the JAK1/JAK2 training sets. LigPrep version 2024-1 (Schrödinger, LLC, New York, NY, USA) [43] was used to generate 3D structures, assign ionization states at pH 7.4, enumerate tautomers where relevant, and minimize geometries using the OPLS force field. Alongside the chemically diverse compounds described above, the library comprised three literature-reported JAK1 inhibitors, with reported selectivity values of approximately 3-fold, 30-fold, and 70-fold (Table S2, Supplementary Materials). These molecules were added to enable an internal control for the prospective screening workflow, and they were processed identically to all other library compounds.

### 3.7. Molecular Mechanics and Interaction Fingerprint Analysis

Docked ligand poses were analyzed to identify key interactions within the binding site, including hydrogen bonds, hydrophobic contacts, and aromatic interactions. Protein–ligand interaction fingerprints (PLIFs) were generated using the Maestro interface of Schrödinger version 2024-1 [48]. The resulting PLIFs were used to systematically compare binding modes across compounds, identify common interaction patterns among actives, and assess selectivity determinants between JAK1 and JAK2.

### 3.8. Residue Numbering Scheme

In the present study, amino-acid positions were assigned according to the KLIFS nomenclature [29], a standardized structure-based numbering system for protein kinases. In this scheme, residues from all structures are mapped onto a kinome-wide multiple sequence and structural alignment, ensuring that functionally equivalent positions are consistently identified across kinases irrespective of their native sequence numbering. This approach enables unambiguous comparison of interaction patterns, conserved motifs, and selectivity-relevant residues across all representative structures used in this study.

### 3.9. ADME-Tox and Drug-Likeness Evaluation

ADME-Tox analysis and drug-likeness evaluation was performed on the prioritized compounds to characterize their pharmacokinetic profiles and development potential. Parameters including gastrointestinal absorption, skin permeation, blood–brain barrier penetration, predicted interactions with cytochrome P450 isoenzymes, toxicity estimates, and synthetic accessibility were computed. Drug-likeness predictions based on the Lipinski, Veber, and Ghose rules were generated using the SwissADME platform [36].

## 4. Conclusions

We integrated large-scale structural analysis, ensemble docking, and interaction fingerprinting to systematically examine the molecular features associated with relative JAK1 selectivity within the JAK kinase family. The results show that explicitly accounting for conformational diversity significantly enhances the ability of docking-based approaches to discriminate active compounds and recover experimentally observed selectivity trends.

Mechanistically, the presented analysis shows that JAK1 selectivity is not driven by unique interactions, but rather by subtle modulation of a conserved binding network. In particular, enhanced van der Waals complementarity and favorable engagement of the Glu aD.55 region, combined with increased local flexibility, emerge as key contributors to preferential ligand recognition. These findings highlight the importance of fine-tuning hydrophobic packing within a highly conserved active site as a viable strategy for achieving kinase selectivity.

Application of the optimized workflow to an external compound library led to the identification of 174 candidates with predicted JAK1 preference, supported by both structural consistency and enrichment of kinase-relevant chemotypes. This demonstrates that the proposed approach is not only mechanistically informative but also effective in prioritizing chemically and biologically relevant candidates.

## Data Availability

The original contributions presented in this study are included in the article/Supplementary Material. Further inquiries can be directed to the corresponding author(s).

## Supplementary Materials

The following supplementary Materials can be downloaded at: https://www.mdpi.com/article/10.3390/ph19050709/s1. Figure S1: JAK1 and JAK2 datasets used in virtual screening workflow; Figure S2: Radar charts illustrating the molecular property profiles of active (yellow) and inactive (blue) inhibitors for JAK1 (A) and JAK2 (B); Figure S3: Interaction fingerprint for filgotinib, itacitinib, and upadacitinib and JAK1 (A) and JAK2 (B) binding site; Figure S4: Hierarchical clustering of JAK1 structures into five clusters based on binding site similarity; Table S1: Summary of clustering results for the JAK1 and JAK2 ensembles; Table S2: JAK1 selectivity data; Table S3: Screening performance evaluation: TP, FP, Precision, Recall, F1-score, and Enrichment Factor (EF) per kinase and method; Table S4: VS output; Table S5: Redocking validation of selected JAK1 and JAK2 complexes: heavy-atom RMSD values relative to crystallographic binding modes; Table S6: PDB codes considered in the study [49].

## Abbreviations

The following abbreviations are used in this manuscript:

RR	Relative Ranking
