# Supplementary material for: Structure-Based Identification of JAK1-Selective Candidates Using Ensemble Docking and Interaction Analysis
Source: Pharmaceuticals (Basel). 2026 Apr 30;19(5):709. doi: 10.3390/ph19050709 (PMC13209238; doi:10.3390/ph19050709)
Supplement: Supplementary file 1 [file pharmaceuticals-19-00709-s001.zip › SupplementaryMaterials_pharmaceuticals.pdf]

Supplementary Materials

for

Structure-Based Identification of JAK1- Selective Candidates Using Ensemble Docking and Interaction Analysis

Nicoleta Stoian, Sorin Avram, Liliana Halip

Table S1 - Summary of clustering results for the JAK1 and JAK2 ensembles

**Cluster**	**Cluster 1**	**Cluster 2**	**Cluster 3**	**Cluster 4**	**Cluster 5**	**Kinase**
**Structures per Cluster**	14	10	34	10	4	JAK1
**Number of Selected Representatives**	3	2	7	2	1	
**Average RMSD**	1.4	0.82	1.31	0.97	0.63	
**RMSD range per Cluster**	0.482-2.28	0.334-1.47	0.28-2.82	0.61-1.76	0.22-1.09	
**Average RMSD among Selected Representatives**	1.14	0.66	1.52	0.77	0	
**RMSD range among Selected Representatives**	0-2	0-1.33	0-2.82	0-1.54	0-0	
**Cluster**	33	22	15	13	35	JAK2
**Structures per Cluster**	7	4	3	3	7	
**Number of Selected Representatives**	1.43	1.38	1.41	1.74	0.5	
**Average RMSD**	0.543-2.214	0.57-2.07	0.47-2.59	0.33-4.07	0.54-0.77	
**RMSD range per Cluster**	1.41	1.05	0.83	2.002	1.03	
**Average RMSD among Selected Representatives**	0.86-2.11	1.41-1.88	1.66-1.66	0.88-4.03	1.42-1.744	

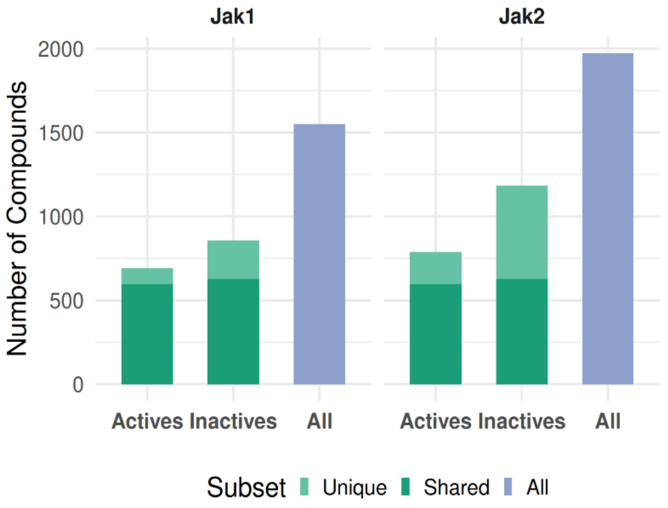

Figure S1 - JAK1 and JAK2 datasets used in virtual screening workflow

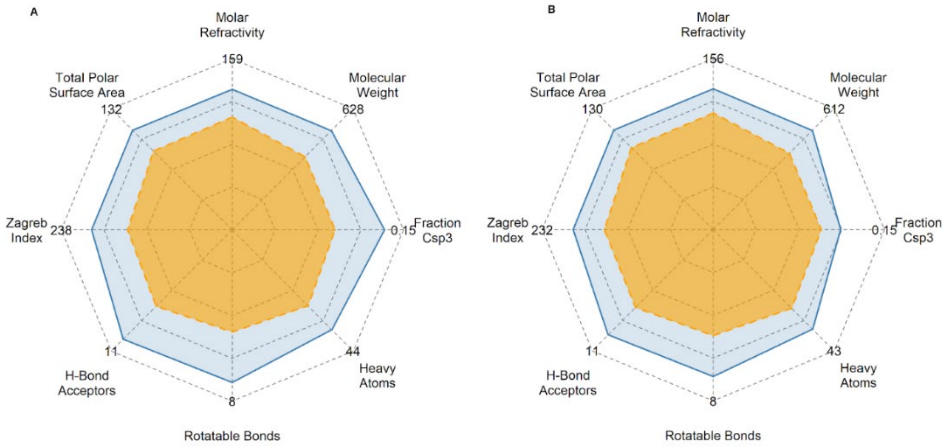

Figure S2. Radar charts illustrating the molecular property profiles of active (yellow) and inactive (blue) inhibitors for JAK1 (A) and JAK2 (B).

Table S2 – JAK1 selectivity data [49]

Name	Compound	JAK1 IC50 (nM)	JAK2 IC50 (nM)
filgotinib	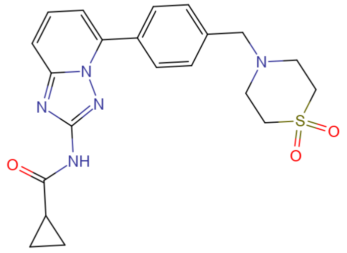	10	28
itacitinib	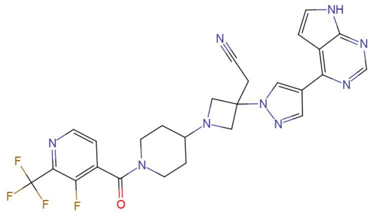	2	63
upadacitinib	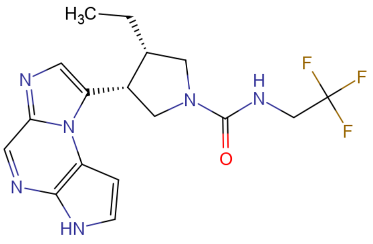	8	600

Table S3. Screening performance evaluation: true positive (TP), false positive (FP), false-negative(FN), true-negative (TN), Precision, Recall, F1-score, and Enrichment Factor (EF) per kinase and method

Kinase	Cutoff	Top_k	TP	FP	FN	TN	Precision	Recall	F1 Score	EF
JAK1	0.2 (%)	3	3	0	705	1586	0.833	0.004	0.007	2.12
	0.5 (%)	8	7	1	701	1580	0.884	0.01	0.02	1.98
	1 (%)	16	14	2	694	1571	0.897	0.02	0.04	1.98
	2 (%)	32	29	3	680	1554	0.895	0.04	0.077	2.05
	5 (%)	79	71	8	637	1502	0.894	0.1	0.18	2.01
	10 (%)	158	137	21	571	1410	0.868	0.194	0.317	1.94
JAK2	0.2 (%)	4	3	1	787	1988	0.719	0.004	0.007	1.9
	0.5 (%)	10	8	2	782	1981	0.817	0.01	0.02	2.03
	1 (%)	20	17	3	773	1970	0.85	0.022	0.042	2.15
	2 (%)	40	34	6	756	1947	0.849	0.043	0.082	2.15
	5 (%)	99	83	16	707	1878	0.837	0.105	0.186	2.1
	10 (%)	198	162	36	629	1759	0.816	0.204	0.327	2.05

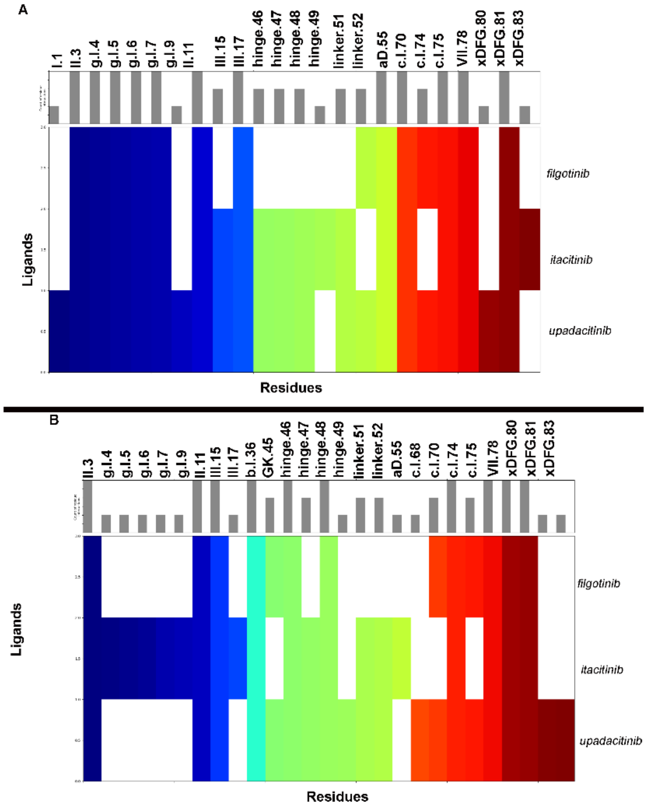

Figure S3 - Interaction fingerprint for filgotinib, itacitinib and upadacitinib and JAK1 (A) and JAK2(B) binding site

Table S5- Redocking validation of selected JAK1 and JAK2 complexes: heavy-atom RMSD values relative to crystallographic binding modes

Kinase	PDB ID	Chain	PDB Ligand ID	ChEMBL ID	rmsd
JAK1	6N77	A	KEJ	CHEMBL2178801	0.87
JAK1	4IVB	A	1J5	CHEMBL2386633	0.54
JAK1	3EYG	A	MI1	CHEMBL2103743	0.39
JAK1	4E4L	E	0NH	CHEMBL2152409	1.56
JAK2	4HGE	B	15V	CHEMBL2178806	1.44

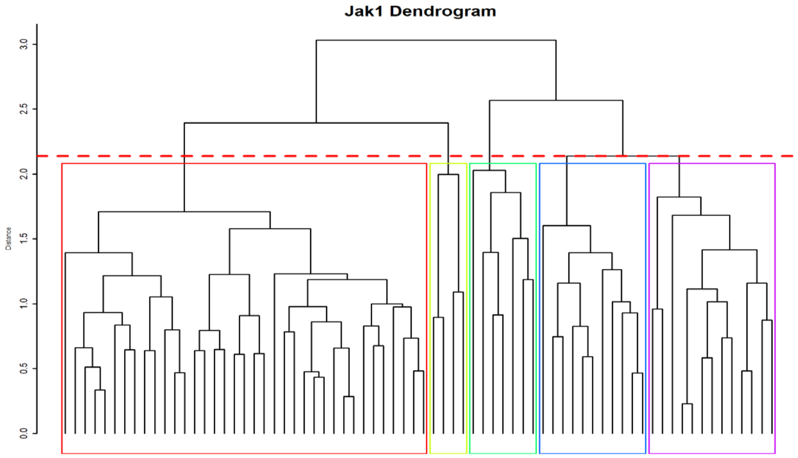

Figure S4 - Hierarchical clustering of JAK1 structures into five clusters based on binding site similarity

Table S6. PDB codes considered in the study

JAK1	JAK2
3eyg, 3eyh, 4e4l, 4e4n, 4e5w, 4ehz, 4ei4, 4fk6, 4i5c, 4ivb, 4ivc, 4ivd, 4k6z, 4k77, 5e1e, 5hx8, 5khw, 5wo4, 6aah, 6bbu, 6c7y, 6dbn, 6elr, 6ggh, 6hzu, 6n77, 6n78, 6n79, 6n7a, 6n7b, 6n7d, 6rsb, 6rsc, 6rsd, 6rse, 6rsh, 6sm8, 6smb, 6tpe, 6tpf, 6w8l	2b7a, 2w1i, 3fup, 3krr, 3lpb, 3q32, 3tjc, 3tjd, 3ugc, 4aqc, 4e4m, 4f08, 4f09, 4gfm, 4gl9, 4gmy, 4hge, 4iva, 4ji9, 4jia, 4p7e, 4yth, 4yti, 4zim, 5aep, 5cf4, 5cf5, 5cf6, 5cf8, 5hez, 5l3a, 5tq3, 5tq4, 5tq5, 5tq6, 5tq7, 5tq8, 5usy, 6bbv, 6tpd, 6vgl, 6vn8, 6vnb, 6vnc, 6vne, 6vnf, 6vng, 6vnh, 6vni, 6vnj, 6vnk, 6vnl, 6vnm, 6vs3, 6vsn, 6wtn, 7ll4, 7ll5, 7q7i, 7q7k, 7q7l, 7q7w, 7uyw, 8bm2, 8bpv, 8bpw, 8bx6, 8bx9, 8bxc, 8bxh, 8g6z, 8g8o, 8g8x
